# Kidney function decline after a non-dialysis-requiring acute kidney injury is associated with higher long-term mortality in critically ill survivors

**DOI:** 10.1186/cc11419

**Published:** 2012-07-12

**Authors:** Chun-Fu Lai, Vin-Cent Wu, Tao-Min Huang, Yu-Chang Yeh, Kuo-Chuan Wang, Yin-Yi Han, Yu-Feng Lin, Ying-Jheng Jhuang, Chia-Ter Chao, Chih-Chung Shiao, Pi-Ru Tsai, Fu-Chang Hu, Nai-Kuan Chou, Wen-Je Ko, Kwan-Dun Wu

**Affiliations:** 1 Department of Internal Medicine, National Taiwan University Hospital and National Taiwan University College of Medicine, 7 Chung Shan S Rd, Taipei 100, Taiwan; 2 Department of Internal Medicine, National Taiwan University Hospital Yun-Lin Branch, 579 Sec 2, Yunlin Rd, Douliou City, Yunlin County 640, Taiwan; 3 Department of Anesthesiology, National Taiwan University Hospital and National Taiwan University College of Medicine, 7 Chung Shan S Rd, Taipei 100, Taiwan; 4 Department of Surgery, National Taiwan University Hospital and National Taiwan University College of Medicine, 7 Chung Shan S Rd, Taipei 100, Taiwan; 5 Department of Traumatology, National Taiwan University Hospital and National Taiwan University College of Medicine, 7 Chung Shan S Rd., Taipei 100, Taiwan; 6 Department of Internal Medicine, Saint Mary's Hospital, 160 Chung-Chen S Rd., Luodong, Yilan County 265, Taiwan; 7 International Harvard Statistical Consulting Company, 7F-11, 57 Chongqing S Rd, Taipei 100, Taiwan

## Abstract

**Introduction:**

The adverse consequences of a non-dialysis-requiring acute kidney injury (AKI) are unclear. This study aimed to assess the long-term prognoses for critically ill patients experiencing a non-dialysis-requiring AKI.

**Methods:**

This retrospective observational cohort study investigated non-dialysis-requiring AKI survivors in surgical intensive care units between January 2002 and June 2010. All longitudinal post-discharge serum creatinine measurements and information regarding end-stage renal disease (ESRD) and death were collected. We assessed the long-term outcomes of chronic kidney disease (CKD), ESRD and all-cause mortality beyond discharge.

**Results:**

Of the 922 identified critically ill patients with a non-dialysis-requiring AKI, 634 (68.8%) patients who survived to discharge were enrolled. A total of 207 patients died after a median follow-up of 700.5 days. The median intervals between the onset of the AKI and the composite endpoints "stage 3 CKD or death", "stage 4 CKD or death", "stage 5 CKD or death", and "ESRD or death" were 685, 1319, 1743, and 2048 days, respectively. This finding shows a steady long-term decline in kidney function after discharge. Using the multivariate Cox proportional hazard model, we found that every 1 mL/min/1.73 m^2 ^decrease from baseline estimated glomerular filtration rate (eGFR) of individuals who progressed to stage 3, 4, and 5 CKD increased the risks of long-term mortality by 0.7%, 2.3%, and 4.1%, respectively (all *p *< 0.05). This result indicates that the mortality risk increased significantly in a graded manner as kidney function declined from the baseline eGFR to advanced stages of CKD during the follow-up period.

**Conclusions:**

In critically ill patients who survive a non-dialysis-requiring AKI, there is a need for continuous monitoring and kidney function protection beyond discharge.

## **Introduction**

Acute kidney injury (AKI) is a major contributor to morbidity and mortality in hospitalized patients [1]. Epidemiological studies have found that there is a gradual increase in the incidence of AKI no matter whether or not the patient requires dialysis [2-4]. Although many studies on patients with AKI who require dialysis have been performed, the literature is rather limited regarding AKI patients who do not require dialysis. Recent reports have indicated that even the smallest changes in serum creatinine (SCr) pose a significant risk for adverse outcomes in AKI patients [5,6]. AKI that does not require dialysis may be of equal or greater importance from a public health perspective than severe AKI requiring dialysis [7]. In addition to disease severity, some AKI patients do not receive dialysis due to physician or patient preferences [3]. Thus, further clinical research is warranted focusing on AKI patients who do not receive dialysis.

The short-term adverse consequences of AKI during acute hospital admission have been well defined [1]. Whereas the long-term risks of end-stage renal disease (ESRD) following AKI have also been determined [8-10], the impact of AKI on the trajectory of decline in kidney function remained poorly defined until only a few years ago. Recent reports have demonstrated that a considerable number of patients with AKI present with only partial renal recovery, suggesting that an AKI is a risk factor for chronic kidney disease (CKD) after discharge [11-15]. However, this issue has yet to be clarified in critically ill survivors. Furthermore, there remains a lack of direct evidence on how the initiation and progression of CKD after an AKI harm the longevity of patients. In fact, this problem is overlooked by both doctors and patients. One report from the United States demonstrated that only a small proportion of AKI survivors, especially those not requiring dialysis, visited a nephrologist within 6 months of hospital discharge [16].

We hypothesized that AKI patients not requiring dialysis also have a high risk for long-term adverse outcomes. To test this hypothesis, this study aimed to investigate the long-term outcomes, specifically those concerning the development or progression of CKD and mortality, among critically ill AKI survivors following hospital discharge. Special attention was also focused on the impact of CKD progression on long-term mortality, which to the best of our knowledge has never been reported.

## Materials and methods

### Study cohort

This study was a retrospective observational cohort study based on the National Taiwan University Hospital Study Group on Acute Renal Failure (NSARF) database established in the surgical intensive care units (ICU) of a medical center and its three branch hospitals in different cities [17-23]. This database contains data prospectively collected from patients with AKI during their stay in ICU and continuously recorded data for outcome analysis. The AKI was defined and classified according to the risk, injury, failure, loss, and end-stage kidney (RIFLE) criteria [24]. In the present study, we enrolled all patients who experienced AKI and survived to discharge but did not receive renal replacement therapy throughout the index hospitalization between January 1 2002 and June 30 2010. Patients were excluded if they had a history of ESRD or kidney transplantation. The follow-up period was continued until December 31 2010.

The study was approved by the Institutional Review Board of National Taiwan University Hospital (No.31MD03). An informed consent was waived because there was no breach of privacy, and the study did not interfere with the clinical decisions related to patient care.

### Clinical assessment of study patients

Baseline demographic and clinical data were assessed at the time of hospital admission, as previously reported [19]. Pertinent medical history included diabetes mellitus (DM, defined as being treated with oral hypoglycemic agents or insulin), hypertension (defined as taking anti-hypertensive drugs or systolic and diastolic blood pressure > 145/95 mmHg at the time of hospitalization), coronary arterial disease (CAD, defined by the diagnosis of ischemic heart disease prior to admission, and positive electrocardiographic findings), cerebral vascular accidents (CVA, ischemic or hemorrhagic), severe congestive heart failure (CHF, defined as a New York Heart Association (NYHA) functional class of III or IV), severe chronic obstructive pulmonary disease (COPD, defined as requiring long-term bronchodilators or steroids), metastatic carcinoma, and hematological malignancies. Body mass index (BMI) was calculated upon admission to the hospital.

All of the clinical parameters and biochemical data were recorded at ICU admission and at the peak of the AKI, including the acute physiology and chronic health evaluation (APACHE) II [25] and sequential organ failure assessment (SOFA) [26] scores. Data were also recorded on the use of inotropic drugs, expressed as inotropic equivalent (mcg/kg/min) = dopamine + dobutamine + 100 × epinephrine + 100 × norepinephrine + 100 × isoprotenolol + 15 × milrinone [27]; mechanical ventilation; cardiopulmonary resuscitation (CPR); intra-aortic balloon pump (IABP); extracorporeal membrane oxygenation (ECMO); total parenteral nutrition (TPN), and any history of surgery (elective or emergent). Body weight change was calculated as the percentage change from ICU admission to the peak of the AKI.

Baseline SCr was determined as the last value measured at least one month, but no more than a year, prior to the index admission [28]. For patients without valid premorbid data, we used the lowest SCr value during the index admission [29]. The estimated glomerular filtration rate (eGFR) was calculated via the Modification of Diet in Renal Disease (MDRD) equation [30]. By using both SCr and urine output criteria, the maximum RIFLE stage throughout the index hospitalization was determined [24].

All of the longitudinal SCr measurements after the index hospitalization and during the follow-up period were collected for each enrolled patient. For every post-discharge SCr measurement, the eGFR was calculated via the MDRD equation [30]. All of the eGFR data were derived from SCr measurements, with the exception of the eGFR at 90 days after the onset of the AKI (post-90d-eGFR). It was suggested that AKI patients should be evaluated for the new onset or worsening of pre-existing CKD at least 3 months after the onset of AKI [31-33]. Thus, we estimated the SCr at 90 days after the onset of AKI via linear interpolation from the two closest SCr measurements before and after the 90-day period from the onset of AKI. The post-90d-eGFR was then calculated from the estimated SCr at 90 days after the onset of AKI. In patients who did not have a SCr measurement at > 90 days after the onset of an AKI, the post-90d-eGFR was characterized as unknown.

### Outcomes

The endpoints included stages 3 to 5 CKD entries, ESRD, and mortality during the follow-up period. Stage 3 to 5 CKD entries were defined as the first day when the eGFR decreased below 60, 30, and 15 mL/min/1.73 m^2^, respectively. ESRD was the certification by a nephrologist of the need for long-term dialysis, as determined from the national Taiwan Renal Registry Data System, which receives data on all dialysis patients every 3 months. Patient survival after discharge was determined from the National Health Insurance Research Database (NHIRD) [34] in February 2011. The NHIRD contains health care data on > 99% of the entire population in Taiwan and covers all inpatient and outpatient medical benefit claims. The survival period was calculated from the onset of AKI until each outcome, whichever came first.

### Statistical analyses

Statistical analyses were performed using Stata 10.0 (StataCorp, College Station, TX, USA) and R 2.11.1 (R Foundation for Statistical Computing, Vienna, Austria). A two-sided *P *value ≤ 0.05 was considered statistically significant. The distributional properties of the continuous variables were presented by either means ± SD or medians (25th, 75th percentiles), whereas categorical variables were presented as frequencies and percentages. The differences between the groups were assessed using the chi square (χ^2^) test, Fisher's exact test, and the two-sample *t*-test, as indicated.

The impact of AKI on the prognoses of the initial CKD or non-CKD patients was carefully evaluated stepwise. First, allowing 3 months as a reasonable period of the time for patients to recover from the AKI [31-33], we considered 90 days after the onset of AKI as the starting point to examine the endpoints of CKD entries, using a Cox proportional hazards model. Specifically, stages 3, 4, and 5 CKD were assessed only in patients with post-90d-eGFR ≥ 60, 30, and 15 mL/min/1.73 m^2^, respectively. Therefore, the patients eligible for the assessment of stage 3 CKD were also eligible for the risk assessments of stage 4 and 5 CKD, and the patients eligible for the risk assessment of stage 4 CKD were also eligible for the risk assessment of stage 5 CKD. In addition, all of the enrolled patients were eligible for the risk assessments of ESRD and long-term mortality after the onset of AKI. The Kaplan-Meier method was applied to estimate survival curves for all the outcomes.

Next, to examine the harmful effects of a CKD entry and ESRD on a patient's time to death, the time-dependent variables of stage 3, 4, and 5 CKD (CKD3, 4, 5-TD), ESRD (ESRD-TD), and the related interaction terms were put into the Cox regression analysis model. Technically, the original wide-form data (n = 634) were reconstructed into a long-form structure (n = 53,833) using the so-called counting process style of input for the specified Cox regression model [35,36]. Then at each ordered event time, we recorded the values of the CKD3-TD, CKD4-TD, CKD5-TD, ESRD-TD, and the related interaction terms for each of the patients at risk in the transformed long-form data set.

The basic model-fitting techniques for (1) stepwise variable selection, (2) goodness-of-fit assessment, and (3) regression diagnostics (for example, the verification of proportional hazards assumption, residual analysis, detection of influential cases, and determination of multicollinearity) were used in our regression analyses.

## Results

Of the 922 patients identified with AKI that did not require dialysis, 634 patients (68.8%) who survived to discharge were enrolled. The ages of the included patients ranged from 16 to 92 years (mean 64.4 ± 15.7 years), and there were 218 women (34.4%) and 182 diabetic patients (28.7%). Thirty-two patients had a history of organ transplantation (excluding renal transplantation); of this group, sixteen patients were liver recipients, fifteen were heart recipients, and one was a lung recipient. With respect to the baseline renal function, 399 patients (62.9%) had a preserved eGFR. The proportions of patients in each maximum RIFLE stage were as follows: 34.9% (n = 221) were in the risk stage, 36.0% (n = 228) were in the injury stage, and 29.2% (n = 185) were in the failure stage. The demographic and clinical characteristics of the study population are presented in Table 1.

**Table 1:** The demographic and clinical characteristics of the included patients (n = 634)

**Demographic data**	
Age, years	64.4 ± 15.7
Women	218 (34.4)
BMI (kg/m^2^)	24.1 ± 4.4
Baseline eGFR (mL/kg/min)	75.7 ± 40.1
Baseline renal function	
preserved eGFR	399 (62.9)
stage 3 CKD	190 (30.0)
stage 4 CKD	38 (6.0)
stage 5 CKD	7 (1.1)
Diabetes	182 (28.7)
Hypertension	370 (58.4)
Smoking	121 (19.1)
CAD	220 (34.7)
CVA	50 (7.9)
Severe CHF*	130 (20.5)
Severe COPD^†^	19 (3.0)
Organ transplantation	32 (5.1)
Metastatic carcinoma	23 (3.6)
Hematological malignancies	3 (0.5)
	
**ICU admission**	
Admission year	
2002 -- 2005	168 (26.5)
2006 - 2010	466 (73.5)
Admitted immediately after surgery	567 (89.4)
CPR	20 (3.2)
IABP	43 (6.8)
ECMO	24 (3.8)
Ventilator	520 (82.0)
TPN	56 (8.8)
	
**Surgery**	
Admission services	
chest surgery	29 (4.6)
cardiovascular surgery	330 (52.1)
neurosurgery	36 (5.7)
general surgery	239 (37.7)
Surgery during admission	576 (90.9)
Emergency surgery	186 (29.3)
	
**Data at the peak of AKI**	
Maximum RIFLE stage^†^	
Risk	221 (34.9)
Injury	228 (36.0)
Failure	185 (29.2)
MAP (mmHg)	89.3 ± 15.7
Body weight change (%)	-0.2 ± 4.5
Hemoglobin (g/dL)	10.9 ± 2.0
Lactate (mmol/L)	2.5 ± 2.5
Creatinine (mg/dL)	2.5 ± 1.3
Urine output (mL/d)	2,057.1 ± 1,220.7
Albumin (g/dL)	3.3 ± 0.7
CVP level (mmHg)	10.3 ± 4.0
Inotropic equivalent (mcg/kg/min)^§^	5.5 ± 14.1
APACHE II score	9.1 ± 5.3
SOFA score	7.2 ± 3.4

Data are presented as the mean ± standard deviation or the number and corresponding percentage (%).*Defined as New York Heart Association functional class III or IV. ^† ^Defined as requiring treatment with long-term bronchodilators or steroids. ^† ^Defined and classified according to the highest RIFLE stage throughout the hospitalization. ^§^Inotropic equivalent (mcg/kg/min) = dopamine + dobutamine + 100 × epinephrine + 100 × norepinephrine + 100 × isoprotenolol + 15 × milrinone. AKI, acute kidney injury; APACHE II, acute physiology and chronic health evaluation II score; BMI, body mass index; CAD, coronary arterial disease; CHF, congestive heart failure; CKD, chronic kidney disease; COPD, chronic obstructive pulmonary disease; CPR, cardiopulmonary resuscitation; CVA, cerebral vascular accident; CVP, central venous pressure; ECMO, extracorporeal membrane oxygenation; eGFR, estimated glomerular filtration rate; IABP, intra-aortic balloon pump; MAP, mean arterial pressure; SOFA, sequential organ failure assessment score; TPN, total parenteral nutrition.

### The development and progression of CKD during the long-term follow-up period

According to the calculated post-90d-eGFR, renal function status at 90 days after the onset of AKI is shown in Table 2A. At that time, only 187 patients (29.5%) had a preserved eGFR, while 244 (38.5%) had CKD. A post-90d-eGFR could not be obtained in the 160 patients (25.2%) who had no SCr measurement at > 90 days after the onset of AKI and in the 43 patients (6.8%) who died before 90 days after the onset of AKI.

**Table 2:** The distribution of renal function status at 90 days following the onset of AKI and the assessment of long-term outcomes

(A) Renal function status at 90 days following the onset of AKI (n = 634)		(B) Eligibility and number of subjects assessed for each long-term outcome (see statistical analyses section)		
**Post-90d-eGFR-based renal function status**	**n**	**Assessment of long-term outcomes**	**Eligible patients**	***n***

(I) Preserved eGFR	187 (29.5%)	Stage 3 CKD	post-90d-eGFR ≥ 60 mL/min/1.73 m^2^	187 (I)
(II) Stage 3 CKD	187 (29.5%)	Stage 4 CKD	post-90d-eGFR ≥ 30 mL/min/1.73 m^2^	374 (I+II)
(III) Stage 4 CKD	47 (7.4%)	Stage 5 CKD	post-90d-eGFR ≥ 15 mL/min/1.73 m^2^	421 (I+II+III)
(IV) Stage 5 CKD	10 (1.6%)	ESRD	all hospital survivors	634 (I+II+III+IV+V+VI)
(V) Unknown	160 (25.2%)	Mortality	all hospital survivors	634 (I+II+III+IV+V+VI)
(VI) Death before 90 days	43 (6.8%)			

AKI, acute kidney injury; CKD, chronic kidney disease; post-90d-eGFR, estimated glomerular filtration rate at 90 days after AKI; preserved eGFR, estimated glomerular filtration rate 60 mL/min/1.73 m^2 ^or higher.

**Figure 1:**
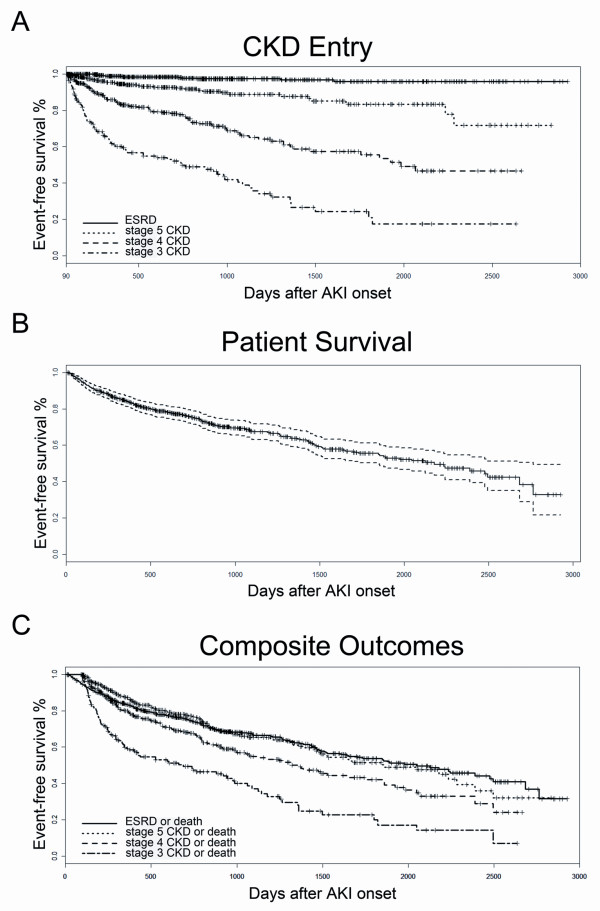
**The Kaplan-Meier survival curve for time (days) from AKI onset to long-term outcomes**. (A) Time to stage 3 to 5 CKD or ESRD; (B) time to all-cause mortality; (C) time to composite outcomes of "stage 3 CKD or death", "stage 4 CKD or death", "stage 5 CKD or death", and "ESRD or death". AKI: acute kidney injury; CKD: chronic kidney disease; ESRD: end-stage renal disease.

The long-term outcomes pertaining to stage 3, 4, and 5 CKD were only assessed in hospital survivors who had a post-90d-eGFR ≥ 60 (n = 187), 30 (n = 374), and 15 mL/min/1.73 m^2 ^(n = 421), respectively (Table 2B). All 634 patients were assessed for the long-term risks of ESRD and mortality (Table 2B). The median time interval between the onset of the AKI and the development of stage 3 and 4 CKD was 740 and 1,984 days, respectively. Only 35 patients (5.5%) developed stage 5 CKD, and 13 patients (2.1%) developed ESRD during the follow-up period (Figure 1A).

### All-cause mortality and composite outcomes during follow-up

The median follow-up duration was 700.5 (330.5, 1,296) days. The endpoint of all-cause mortality occurred in 207 patients (32.7%). The Kaplan-Meier curves showed that the median patient survival time was 2,178 days after the onset of AKI (Figure 1B).

The development of advanced CKD and mortality are competitive endpoints, as many AKI survivors do not live long enough to develop advanced CKD. We therefore assessed the composite outcomes. The median survival time of patients assessed for the composite endpoints "stage 3 CKD or death", "stage 4 CKD or death", "stage 5 CKD or death", and "ESRD or death", were 685, 1,319, 1,743, and 2,048 days following the onset of AKI, respectively (Table 3 and Figure 1C).

**Table 3:** The observations for composite endpoints

Composite outcome	Eligible patients (number)	Observation period* (days)	**Outcome events**^† ^			Median event-free survival time (days)
				
			CKD-entry events before death	Death events before CKD outcome	Composite events	
Stage 3 CKD or death	187	306 (178.5, 763.5)	93 (49.7)	10 (5.3)	103 (56.3)	685
Stage 4 CKD or death	374	509.5 (258.2, 957)	92 (24.6)	50 (13.4)	142 (38.0)	1319
Stage 5 CKD or death	421	631 (298, 1074)	35 (8.3)	91 (21.6)	126 (29.9)	1743
ESRD or death	634	692.5 (309.8, 1285)	13 (2.1)	202 (31.9)	215 (33.9)	2,048

*Observation period is presented as the median (25th, 75th percentile); ^† ^event is presented as the number and corresponding percentage (%)

CKD: chronic kidney disease; ESRD: end-stage renal disease.

### Risk factors for long-term outcomes

A Cox proportional hazards model, adjusted for all of the variables listed in Table 1 including baseline eGFR, was conducted to identify the independent factors associated with each composite outcome (Table 4). It was found that age was consistently a risk factor for all of the composite endpoints. A higher baseline eGFR was protective against the composite outcomes of "stage 3 CKD or death" and "stage 4 CKD or death". When assessing the composite outcomes of "stage 4 CKD or death" and "stage 5 CKD or death", SCr at the peak of the AKI appeared to be an important risk factor.

**Table 4:** A multivariate Cox proportional hazards model for independent factors associated with the composite outcomes

Composite outcome	Covariate	HR (95% CI)	P
Stage 3 CKD or death	baseline eGFR	0.99 (0.98, 1.00)	0.02
(n = 187)	age	1.02 (1.00, 1.03)	0.02

Stage 4 CKD or death	received surgery	0.42 (0.25, 0.72)	0.002
(n = 374)	hypertension	0.56 (0.39, 0.80)	0.002
	baseline eGFR	0.99 (0.98, 1.00)	0.009
	age	1.04 (1.02, 1.06)	< 0.001
	SCr at the peak of AKI	1.20 (1.04, 1.38)	0.01
	diabetes	1.79 (1.25, 2.58)	0.002
	IABP	1.88 (1.14, 3.09)	0.01

Stage 5 CKD or death	organ transplantation	0.30 (0.09, 0.96)	0.04
(n = 421)	BMI	0.94 (0.90, 0.99)	0.02
	age	1.04 (1.03, 1.06)	< 0.001
	SCr at the peak of AKI	1.20 (1.05, 1.36)	0.006
	general surgical services	2.09 (1.45, 3.01)	< 0.001
	CPR	3.22 (1.36, 7.62)	0.008

AKI, acute kidney injury; BMI, body mass index; CI, confidence interval; CPR, cardiopulmonary resuscitation; eGFR, estimated glomerular filtration rate; HR, hazard ratio; IABP, intra-aortic balloon pump; SCr, serum creatinine.

Additionally, another Cox proportional hazards model, adjusted for all of the variables listed in Table 1, including baseline eGFR, was performed to evaluate the independent factors associated with long-term mortality. Time-dependent CKD3, 4, 5-TD, ESRD-TD, and the related interaction terms were also included in the regression model. After adjusting for the effects of the other covariates in the final model, the interaction terms of baseline eGFR and CKD3, 4, and 5-TD, but not ESRD-TD, significantly predicted long-term mortality (Table 5). The main CKD3, 4, and 5-TD factors themselves were not significant in the final model. The hazard ratios (HRs) product suggested a 0.7%, 2.3% (1.007*1.016 = 1.023), and 4.1% (1.023*1.017 = 1.041) increase in mortality risk for every 1 mL/min/1.73 m^2 ^decrease from baseline eGFR in individuals who progressed to stage 3, 4, and 5 CKD during the long-term follow-up period, respectively. In other words, among patients with the same baseline kidney function, those who progressed to more advanced CKD had a higher risk of death. Furthermore, among the patients who progressed to the same stage of CKD during the follow-up period, those who had a higher baseline eGFR had a higher risk of death. This indicated that mortality risk increased significantly in a graded manner with kidney function decline from baseline eGFR to the advanced stages of CKD during the follow-up period. The other risk factors for long-term mortality included age (HR 1.04), receiving general surgical services (HR 1.77), and the administration of CPR during ICU admission (HR 6.55). Conversely, patients who received organ transplantation (HR 0.29) and who had a higher mean arterial pressure at the peak of the AKI (HR 0.98) appeared to be at less of a risk for mortality during the follow-up period. The interaction term of CPR and ECMO indicating a higher grade of life support during resuscitation, also predicted a lower risk of death after discharge (HR 0.17). This final model passed the test for proportional hazards assumption and fit the data well (adjusted *R*^2 ^= 0.289).

**Table 5:** A multivariate Cox proportional hazards model with time-dependent variables for the independent factors of long-term mortality (N = 53,833)

Covariate	HR (95% CI)	*P*
**Demographic data**		
organ transplantation	0.29 (0.09, 0.98)	0.05
age	1.04 (1.02, 1.05)	< 0.001
**ICU admission and surgery**		
CPR*ECMO*	0.17 (0.03, 0.99)	0.048
MAP at the peak of AKI	0.99 (0.97, 1.00)	0.006
general surgical services	1.77 (1.19, 2.64)	0.005
receive CPR	6.55 (2.34, 18.32)	< 0.001
Time-dependent variables during follow-up*^† ^		
preserved eGFR during follow-up	1	reference
baseline eGFR*CKD3-TD	1.007 (1.001, 1.012)	0.016
baseline eGFR*CKD4-TD	1.016 (1.010, 1.021)	< 0.001
baseline eGFR*CKD5-TD	1.017 (1.008, 1.026)	< 0.001
baseline eGFR*ESRD-TD	1.054 (0.959, 1.158)	0.3

AKI, acute kidney injury; CI, confidence interval; CKD, chronic kidney disease; CPR, cardiopulmonary resuscitation; ECMO, extracorporeal membrane oxygenation; eGFR, estimated glomerular filtration rate; ESRD, end-stage renal disease; HR, hazard ratio; MAP, mean arterial pressure. *CPR*ECMO indicates the interaction term of receiving both CPR and ECMO; Baseline eGFR*CKD3 (4, 5)-TD indicates the interaction term of baseline eGFR and stage 3 (4, 5) CKD time-dependent variables; Baseline eGFR*ESRD-TD indicates the interaction term of baseline eGFR and ESRD. ^† ^The main CKD3, 4, 5-TD factors were not significant in the final model.

## Discussion

Studies of AKI that does not require dialysis are relatively scant, despite the increasing prevalence of patients with this condition [2,3]. This may be attributed to the difficulty of identifying such cases with diagnosis codes [3,37]. In the present study, we identified AKI patients based on their changes in SCr and followed their every available SCr measurement after their hospital discharge. This enabled us to study the long-term outcomes of CKD and mortality in critically ill patients with AKI that did not require dialysis.

In the past, it was generally accepted that AKI survivors had good renal outcomes, as assessed by a low incidence of ESRD [38,39]. However, this assumption was recently challenged [15]. In one study, it was found that 72.1% of older patients without pre-existing renal failure developed CKD within 2 years of AKI [8]. Two separate large cohort studies both demonstrated that AKI is an independent predictor of the development of advanced CKD [11,12]. Ishani *et al*. also reported that the magnitude of the increase in SCr after cardiac surgery was associated with an increasing long-term risk of incident CKD, progression of CKD, and mortality [14]. Last, another analysis in a cohort undergoing coronary angiography revealed a quicker eGFR decline following an episode of AKI [13].

Patients admitted into the ICU have a higher rate of early mortality than other populations investigated in this field of research [15]. Consequently, clinicians may pay more attention to the risk of death rather than the progression to advanced CKD. Our findings extend the knowledge of this issue to critically ill patients, where a steady deterioration of kidney function was observed in AKI survivors (Figure 1 and Table 3). A subset of patients in our cohort demonstrated a short-term recovery of kidney function and had a post-90d-eGFR ≥ 60 mL/min/1.73 m^2 ^with a median of 75.3 (66.1, 92.5). Approximately half of these patients (49.7%) progressed to CKD during the long-term follow-up period (Table 3). Among these patients, the median interval prior to developing stage 3 CKD was approximately 2 years (Figure 1A) and was even shorter for the composite endpoint of "stage 3 CKD or death" (Figure 1C). Regarding advanced CKD, the median interval from the onset of AKI was less than 4 years to reach the composite outcome of "stage 4 CKD or death", and less than 6 years to reach "ESRD or death" (Figure 1C). These data indicate that we should not view AKI only as a self-limiting acute disease but also as a long-lasting progressive disorder.

The predictive value of the interaction term of baseline eGFR and CKD entry for long-term mortality is a novel finding. In the present study, we collected detailed patient demographic and clinical data during episodes of AKI, including the APACHE II and SOFA scores. This enabled us to adjust the patients' characteristics, baseline renal function, and severity of acute illness to evaluate the effect of CKD progression on long-term mortality. We demonstrated that with a higher baseline kidney function and more advanced CKD progression, there is a greater risk of death (Table 5). This observation suggests that a gradual decline in long-term renal function in non-dialysis-requiring AKI survivors not only reflects kidney dysfunction, but also survival. Thus, one potential modifiable factor for improving the long-term survival of AKI hospital survivors is to prevent the deterioration of their renal function.

Interestingly, the identified risk factors presented in Table 4 are not the same across each long-term composite outcome. This may be due to the different eligibilities of patients accessed for different endpoints. Given that this was a retrospective observational study, we cannot confirm the causal relationship of these variables with progressive renal failure. Besides, organ transplant is found to be an independent protective factor for stage 5 CKD and mortality (Table 4 and 5). These recipients were selected for transplantation before surgery and followed up more carefully after discharge. In contrast, patients receiving general surgical services were more likely to have stage 5 CKD or to have died during the long-term follow-up period (Table 4 and 5). Most non-general surgical patients in our cohort received cardiovascular surgical services. The majority of kidney damage in cardiovascular surgical patients resulted from cardiopulmonary bypass-related ischemia-reperfusion injury. This type of AKI may have a better prognosis than septic AKI [40]. Furthermore, there were 33.9% of AKI patients receiving general surgical services reached the maximum RIFLE stage of failure, in comparison to the 26.3% of other AKI patients (*P *= 0.04).

Our study had limitations. First, 25.2% of the hospital survivors did not have any SCr measurements beyond the 90-day period after AKI onset, and 6.8% of the hospital survivors died before that interval. Those patients without long-term follow-up of kidney function were more likely to be women and to have a history of CVA, and less likely to receive organ transplantation and to use TPN (Additional file 1). Despite this, the linking of this study with the national registry database on ESRD and death provides accurate information regarding these two endpoints. Second, only critically ill patients with AKI not requiring dialysis were studied; as a result, we are unable to generalize our findings to more severe AKI requiring dialysis or to other populations. Comparisons among patients with AKI, who did or did not require dialysis, and CKD without superimposed AKI, also could not be made in this study. Furthermore, the relative risk for CKD or the long-term mortality brought on by AKI could not be determined in this study due to a lack of appropriate non-AKI controls. Despite these limitations, other studies in the literature that provide this information also have similar findings [10-15]. Third, the status of the baseline proteinuria, presence of sepsis, and etiology of AKI were not identified. Recently, these variables were identified as important factors affecting short- and long-term prognosis after AKI [21,40,41]. In this study, we included all available demographic data and clinical information for analysis, but residual confounding might still exist to some extent due to unmeasured and unknown risk factors. Last, this retrospective observational study cannot prove that AKI plays a causal role in CKD progression or that aggressive follow-up after discharge would improve these long-term outcomes, as there was no pre-specified protocol for post-discharge care and SCr follow-up. Prospective studies with structured, multidisciplinary, follow-up medical care for AKI survivors are warranted to clarify this issue [42].

## Conclusions

The present study demonstrated the gradual decline in kidney function after hospital discharge in critically ill patients with AKI, who did not require dialysis. Furthermore, there appeared to be an association between the deterioration of kidney function and long-term mortality. This finding underscores the need to explore the mechanisms that are responsible for the long-term consequences of an AKI. Additionally, there is a need for novel strategies that focus on retarding renal function deterioration and strengthening the continuous surveillance of kidney function in AKI survivors.

## Key messages

• Long-term trajectories of kidney function after AKI in critically ill survivors are not well-defined, especially in those who have not received dialysis. This issue is possibly overlooked in post-discharge medical care.

• A considerable portion of critically ill patients, who survived AKI that did not require dialysis, had CKD during long-term follow-up.

• We revealed that there was a steady long-term decline in kidney function after hospital discharge. Therefore, we should not view AKI only as a self-limiting acute disease, but also as a long-lasting progressive disorder.

• The mortality risk increased significantly in a graded manner with the decline in kidney function from baseline eGFR to advanced stages of CKD during the follow-up period.

• We need to organize multidisciplinary medical care for critically ill AKI survivors to continually monitor kidney function after discharge. One potential modifiable factor to improve long-term survival of patients with AKI after discharge from hospital is to prevent the deterioration of their kidney function.

## Abbreviations

AKI: acute kidney injury; APACHE: acute physiology and chronic health evaluation; BMI: body mass index; CAD: coronary arterial disease; CHF: congestive heart failure; CKD: chronic kidney disease; COPD: chronic obstructive pulmonary disease; CPR: cardiopulmonary resuscitation; CVA: cerebral vascular accident; DM: diabetes mellitus; ECMO: extracorporeal membrane oxygenation; eGFR: estimated glomerular filtration rate: ESRD: end-stage renal disease; HR: hazard ratio; IABP: intra-aortic balloon pump; ICU: intensive care unit; MDRD: Modification of Diet in Renal Disease; NHIRD: National Health Insurance Research Database; NYHA: New York Heart Association; post-90d-eGFR, estimated glomerular filtration rate at 90 days after AKI; RIFLE: risk, injury, failure, loss, and end-stage kidney; SCr: serum creatinine; SOFA: sequential organ failure assessment; TPN: total parenteral nutrition.

## Competing interests

The authors declare that they have no competing interests.

## Authors' contributions

CFL conceived the study, participated in data collection, performed statistical analysis, interpreted results, and wrote the manuscript. VCW, TMH, YFL, YJJ, CTC, and CCS participated in data collection and manuscript revision. YCY, KCW, YYH, PRT, and NKC participated in data collection. FCH performed statistical analysis, participated in data interpretation and manuscript writing. WJK and KDW conceived the study and participated in manuscript revision. All authors read and approved the final manuscript.

## Supplementary Material

Additional file 1 **A table showing the demographic and clinical characteristics of survivors stratified by follow-up duration**. Click here for file
