# Supplementary material for: Kidney function decline after a non-dialysis-requiring acute kidney injury is associated with higher long-term mortality in critically ill survivors
Source: Crit Care. 2012 Jul 12;16(4):R123. doi: 10.1186/cc11419 (PMC3580702; doi:10.1186/cc11419)
Supplement: Additional file 1 — A table showing the demographic and clinical characteristics of survivors stratified by follow-up duration. [file cc11419-S1.DOC]

**Additional file 1.**

**Table S1.** The demographic and clinical characteristics of survivors stratified by follow-up duration

|  | **All Survivors**  (*n*=634) | **F/U < 90days***  (*n*=203) | **F/U > 90days**  (*n*=431) | ***p***^†^ |
| --- | --- | --- | --- | --- |
| **Demographic data** |  |  |  |  |
| age (years) | 64.4 ± 15.7 | 65.1 ± 16.4 | 64.1 ± 15.4 | 0.5 |
| women | 218 (34.4) | 82 (40.4) | 136 (31.6) | 0.03 |
| BMI (kg/m^2^) | 24.1 ± 4.4 | 24.2 ± 4.8 | 24.0 ± 4.2 | 0.7 |
| baseline eGFR (mL/kg/min) | 75.7 ± 40.1 | 76.3 ± 35.8 | 75.5 ± 42.0 | 0.8 |
| baseline renal function  preserved eGFR  stage 3 CKD  stage 4 CKD  stage 5 CKD | 399 (62.9)  190 (30.0)  38 (6.0)  7 (1.1) | 132 (65.0)  55 (27.1)  13 (6.4)  3 (1.5) | 267 (62.0)  135 (31.3)  25 (5.8)  4 (0.9) | 0.6 |
| diabetes | 182 (28.7) | 68 (33.5) | 114 (26.5) | 0.07 |
| hypertension | 370 (58.4) | 121 (59.6) | 249 (57.8) | 0.7 |
| smoking | 121 (19.1) | 37 (18.2) | 84 (19.5) | 0.7 |
| CAD | 220 (34.7) | 67 (33.0) | 153 (35.5) | 0.6 |
| CVA | 50 (7.9) | 23 (11.3) | 27 (6.3) | 0.03 |
| severe CHF^†^ | 130 (20.5) | 36 (17.7) | 94 (21.8) | 0.2 |
| severe COPD^‡^ | 19 (3.0) | 6 (3.0) | 13 (3.0) | 0.9 |
| organ transplantation | 32 (5.1) | 1 (0.5) | 31 (7.2) | <0.001 |
| metastatic carcinoma | 23 (3.6) | 7 (3.5) | 16 (3.7) | 0.9 |
| hematological malignancies | 3 (0.5) | 1 (1.0) | 1 (0.2) | 0.2 |
|  |  |  |  |  |
| **ICU admission** |  |  |  |  |
| admission year  2002 – 2005  2006 - 2010 | 168 (26.5)  466 (73.5) | 60 (29.6)  143 (70.4) | 108 (25.1)  323 (74.9) | 0.2 |
| admitted immediately after surgery | 567 (89.4) | 182 (89.7) | 385 (89.3) | 0.9 |
| CPR | 20 (3.2) | 6 (3.0) | 14 (3.3) | 0.8 |
| IABP | 43 (6.8) | 12 (5.9) | 31 (7.2) | 0.5 |
| ECMO | 24 (3.8) | 7 (3.5) | 17 (3.9) | 0.8 |
| ventilator | 520 (82.0) | 168 (82.8) | 352 (81.7) | 0.7 |
| TPN | 56 (8.8) | 9 (4.4) | 47 (10.9) | 0.007 |
|  |  |  |  |  |
| **Surgery** |  |  |  |  |
| admission services  chest surgery  cardiovascular surgery  neurosurgery  general surgery | 29 (4.6)  330 (52.1)  36 (5.7)  239 (37.7) | 10 (4.9)  110 (54.2)  17 (8.4)  66 (32.5) | 19 (4.4)  220 (51.0)  19 (4.4)  173 (40.1) | 0.1 |
| surgery during admission | 576 (90.9) | 184 (90.6) | 392 (91.0) | 0.9 |
| emergency surgery | 186 (29.3) | 68 (33.5) | 118 (27.4) | 0.2 |
|  |  |  |  |  |
| **Data at the peak of AKI** |  |  |  |  |
| maximum RIFLE stage^§^  Risk  Injury  Failure | 221 (34.9)  228 (36.0)  185 (29.2) | 76 (37.4)  70 (34.5)  57 (28.1) | 145 (33.6)  158 (36.7)  128 (29.7) | 0.6 |
| MAP (mmHg) | 89.3 ± 15.7 | 90.2 ± 15.1 | 88.9 ± 16.0 | 0.3 |
| body weight change (%) | -0.2 ± 4.5 | 0.1 ± 3.7 | -0.3 ± 4.8 | 0.3 |
| hemoglobin (g/dL) | 10.9 ± 2.0 | 11.0 ± 2.1 | 10.9 ± 1.9 | 0.4 |
| lactate (mmol/L) | 2.5 ± 2.5 | 2.5 ± 2.5 | 2.5 ± 2.5 | 0.9 |
| creatinine (mg/dL) | 2.5 ± 1.3 | 2.4 ± 1.4 | 2.5 ± 1.2 | 0.4 |
| urine output (mL/d) | 2057.1 ± 1220.7 | 1969.4 ± 1159.4 | 2099.0 ± 1248.0 | 0.2 |
| albumin (g/dL) | 3.3 ± 0.7 | 3.3 ± 0.7 | 3.3 ± 0.7 | 0.9 |
| CVP level (mmHg) | 10.3 ± 4.0 | 10.6 ± 3.9 | 10.1 ± 4.0 | 0.2 |
| inotropic equivalent (mcg/kg/min)^¶^ | 5.5 ± 14.1 | 6.0 ± 21.1 | 5.3 ± 9.0 | 0.5 |
| APACHE II | 9.1 ± 5.3 | 9.1 ± 5.6 | 9.1 ± 5.2 | 0.9 |
| SOFA | 7.2 ± 3.4 | 7.2 ± 3.5 | 7.2 ± 3.3 | 0.9 |

Data are presented as the mean ± standard deviation or the number and corresponding percentage (%). AKI, acute kidney injury; APACHE II, acute physiology and chronic health evaluation II score; BMI, body mass index; CAD, coronary arterial disease; CHF, congestive heart failure; CKD, chronic kidney disease; COPD, chronic obstructive pulmonary disease; CPR, cardiopulmonary resuscitation; CVA, cerebral vascular accident; CVP, central venous pressure; ECMO, extracorporeal membrane oxygenation; eGFR, estimated glomerular filtration rate; IABP, intra-aortic balloon pump; MAP, mean arterial pressure; SOFA, sequential organ failure assessment score; TPN, total parenteral nutrition.

* Include 160 patients who had no SCr measurements at > 90 days after the onset of the AKI and 43 patients who died before that interval.

^†^ Values represent comparisons between the hospital survivors and non-survivors groups. Two-sample t-tests were used for the comparison of continuous variables.χ^2^ tests or Fisher’s exact tests were used for comparison of categorical variables.

^‡^ Defined as New York Heart Association functional class III or IV.

^§^ Defined as requiring treatment of long-term bronchodilators or steroids.

^||^ Defined and classified according to the highest RIFLE stage throughout the hospitalization.

^¶^ Inotropic equivalent (mcg/kg/min) = dopamine + dobutamine + 100×epinephrine + 100×norepinephrine + 100×isoprotenolol + 15×milrinone.
